# DEVELOPING A COMMUNITY-BASED COMPREHENSIVE FALLS PREVENTION PROGRAM IN COLLABORATION WITH PRIMARY CARE

**DOI:** 10.1093/geroni/igz038.2831

**Published:** 2019-11-08

**Authors:** Dawna Pidgeon

**Affiliations:** Dartmouth-Hitchcock Medical Center, Lebanon, New Hampshire, United States

## Abstract

Reducing falls in older adults requires a comprehensive screening program, a systems approach to refer those at risk and an evidence based community falls prevention programs. The Dartmouth Centers for Healthy & Aging has been the recipient of 2 Association of Community Living (ACL) Falls Prevention grants. This has enabled the development of a robust program for falls screening both in primary care and through community based balance screening events called “Balance Days”. At risk individuals receive coaching, based on the principles of motivational interviewing, focusing on enrolling in either “Matter of Balance” or “Tai Chi Quan: Moving for Better Balance”. Through the ACL grant we have built significant capacity across New England for these programs. This talk will focus on the “secret sauce” of implementing a robust community based falls prevention program in partnership with primary care.

